# Power Equation for Predicting the Risk of Central Nervous System Oxygen Toxicity at Rest

**DOI:** 10.3389/fphys.2020.01007

**Published:** 2020-08-17

**Authors:** Ben Aviner, Ran Arieli, Alexandra Yalov

**Affiliations:** ^1^The Israel Naval Medical Institute, Israel Defense Forces Medical Corps, Haifa, Israel; ^2^Eliachar Research Laboratory, Western Galilee Medical Center, Nahariya, Israel; ^3^HP – Indigo Division, Nes Ziona, Israel

**Keywords:** hyperbaric oxygen treatment, diving, algorithm, convulsions, saturation

## Abstract

Patients undergoing hyperbaric oxygen therapy and divers engaged in underwater activity are at risk of central nervous system oxygen toxicity. An algorithm for predicting CNS oxygen toxicity in active underwater diving has been published previously, but not for humans at rest. Using a procedure similar to that employed for the derivation of our active diving algorithm, we collected data for exposures at rest, in which subjects breathed hyperbaric oxygen while immersed in thermoneutral water at 33°C (*n* = 219) or in dry conditions (*n* = 507). The maximal likelihood method was employed to solve for the parameters of the power equation. For immersion, the CNS oxygen toxicity index is K_*I*_ = t^2^ × PO_2_^10.93^, where the calculated risk from the Standard Normal distribution is Z_*I*_ = [ln(K_*I*_^0.5^) – 8.99)]/0.81. For dry exposures this is K_*D*_ = t^2^ × PO_2_^12.99^, with risk Z_*D*_ = [ln(K_*D*_^0.5^) – 11.34)]/0.65. We propose a method for interpolating the parameters at metabolic rates between 1 and 4.4 MET. The risk of CNS oxygen toxicity at rest was found to be greater during immersion than in dry conditions. We discuss the prediction properties of the new algorithm in the clinical hyperbaric environment, and suggest it may be adopted for use in planning procedures for hyperbaric oxygen therapy and for rest periods during saturation diving.

## Introduction

Patients undergoing hyperbaric oxygen (HBO) therapy and divers engaged in underwater activity breathe pure oxygen at greater than atmospheric pressure. In such situations, there is always an imminent risk of central nervous system oxygen toxicity (CNS-OT). Symptoms range from hearing and vision disturbances to vomiting, dizziness, muscle twitching, convulsions, and loss of consciousness (Donald, 1992; Harabin, 1993). We previously proposed the power equation and exponential recovery algorithm to predict the risk of CNS-OT in an active diver expending energy at 4.4 metabolic equivalents of task (MET) (Arieli et al., 2002). In further studies (Arieli, 2003, 2019), we elaborated ways of alleviating the risk.

The power equation for underwater activity takes the form:

(1) $$K=t^{2}{\mathrm{PO}}_{2}^{6.8}$$

where K is the index of CNS-OT (*CNS-OT index*), t is the time in min, and PO_2_ is the oxygen pressure in bar. When the *CNS-OT index* reaches a critical value Kc, toxicity may appear.

Recovery of the *CNS-OT index* (Krec) was calculated by the equation:

(2) $$\mathrm{Krec}=K\times e^{-0.079\mathrm{trec}}$$

where trec is the recovery time in min. The risk of CNS-OT may then be derived from the Normal distribution of the *CNS-OT index*:

(3) $$Z_{4.4}=\left[\ln{\left(K\right)}^{0.5}-9.63\right]/2.02$$

In contrast to diving, most exposures at 1 MET are conducted in dry conditions in the hyperbaric chamber. There have been claims that immersion carries a higher risk of oxygen toxicity than dry exposure (Donald, 1992; van Ooij et al., 2011; Ciarlone et al., 2019). Underwater exposures are generally conducted at an environmental temperature which drives up the metabolic rate. It therefore seems necessary to clarify the distinction between the effects of metabolic rate and the dry or immersed state. We have previously shown, in the power equation for the rat, that as metabolic rate increases there is a linear decrease in the power of the PO_2_ and in ln(K_*c*_) (Arieli, 2003). The question arises as to whether this may be extrapolated to humans. If this is indeed the case, knowing two points on the metabolic scale, one may then calculate the parameters for any other level of metabolic rate.

However, no algorithm has been developed for the risk of CNS-OT in humans at rest (1 MET). A large number of hyperoxic-hyperbaric exposures are conducted in resting conditions, and for that reason, as we recently suggested (Arieli, 2020), such an algorithm is sorely needed. In our report, we explained the advantage of an air break between oxygen sessions, and how the recovery function may be used to calculate the appropriate time for this break. Formulating the power equation for 1 MET, together with the recovery function, may enable more precise planning of exposures such as clinical treatment in the hyperbaric chamber, and the long stay in an underwater habitat, a saturation diving system on the surface, or a diving bell, among other applications.

## Materials and Methods

### Data Derivation

Data for hyperoxic exposures at rest in immersion or in dry conditions – exposure time, partial pressure of oxygen (PO_2_), and appearance or absence of CNS-OT, were extracted from a number of studies compiled by Harabin (1993), and from Koch et al. (2008). Evidently there are ample data on hyperoxic exposures at rest which were not reported in a proper way for our analysis (exact condition for each single exposure). This is the best model science can generate at this moment with the limited data available. We considered only immersion in thermoneutral water (32.8 ± 1.2°C), and thus a metabolic rate of 1 MET. The time scale was 4–120 min in the immersed state, and 6–180 min in dry conditions. PO_2_ ranged from 2.26 to 3.24 bar in immersion, and from 2.55 to 3.67 bar in dry exposures. The data compiled by Harabin (1993) do not contain demographic information such as age, sex, or state of health. However, because these were gathered from naval oriented studies, we believe that the subjects were healthy males. Koch et al. (2008) state that their data were from healthy, elite Navy combat divers. As in our previous study (Arieli et al., 2002), we selected the symptoms suggested by Harabin et al. (1995) as indicating a positive finding of CNS-OT: nausea, numbness, dizziness, twitching, hearing and visual disturbances, convulsions and unconsciousness. Symptoms were noted in 105 of the 219 immersed, and in 136 of the 507 dry exposures (Table 1).

**TABLE 1:** Hyperoxic exposures compiled from Harabin (1993) andKoch et al. (2008), and used in the present analysis.

Immersed/Dry	PO_2_, bar	Time, minmean (*SD*)	*n*	CNS-OT
Immersed	4.08	18.4 (12.5)	13	11
	3.44	33.0 (23.0)	51	46
	2.83	57.2 (22.8)	155	48
Dry	2.83	117.6 (17.0)	17	14
	3.44	62.1 (36.0)	54	52
	4.08	15.8 (6.7)	8	1
	2.83	122.0 (45.4)	6	5
	3.74	28.4 (20.8)	36	36
	4.05	26.3 (11.2)	17	17
	2.8	29.9 (1.0)	369	11

*PO_2_, partial pressure of oxygen; SD, standard deviation; CNS-OT, Number of subjects presenting central nervous system oxygen toxicity.*

### Statistical Evaluation

To derive parameters for the power equation, a survival parametric regression model was fitted to the meta data (Appendix). We applied the maximum likelihood method to solve for the parameters in this model, as in our previous report (Arieli et al., 2002). We also applied the Wald test and the maximum likelihood ratio test for equality/inequality of immersed to dry exposures.

## Results

There was no difference in either the cumulative probability for “life distribution” between data for 1946 in Harabin (1993) and the data from Koch et al. (2008), or in the fitted risk using the final model as a function of the risk estimated by the individual models for each study. Thus the data from these two reports may be combined.

Both the Wald test (*p* < 0.001) and the maximum likelihood ratio test (*p* < 0.001) indicated inequality between immersed and dry conditions, demonstrating a greater risk in immersion. We therefore chose to separate immersed from dry exposures when solving for the parameters of the power equation.

One group of 14 exposures (PO_2_ = 2.54 bar, time 120 min), in which there were no symptoms of CNS-OT and which thus differed from all the other groups in the goodness-of-fit assessment, was excluded from the analysis. One possibility may be that these were well selected and oxygen-adapted divers.

The distribution fitted to Z is Standard Normal, which was found to be the best fit. The population standard deviation – σ of Normal distribution fitted to the **ln(t)** of survival times from the diverse experiments, was quite different for the various data sets. It may well be that the conglomeration of different groups of subjects in a diversity of experimental setups and countries was the underlying cause of this effect. No specific trend was observed for this variation, and on the assumption that it would not change over all values of PO_2_, we averaged this σ for our model. We observed previously, both in rats (Arieli et al., 2005) and in divers (Arieli et al., 2006), that individual sensitivity to CNS-OT remains the same at different PO_2_s.

The outcome of the analysis is given in Table 2. For immersed exposures, the power equation and the critical *CNS-OT index* are:

(4) $$K_{I}=t^{2}\times {\mathrm{PO}}_{2}^{10.93}$$

$${\mathrm{Kc}}_{I}=6.42\times 10^{7}$$

**TABLE 2:** Solution for the parameters of the power equation Kc = t^2^PO_2_^*c*^.

Approximate parameter distribution	Dry/Immersed	Term	Estimate	SE	Lower 95%	Upper 95%
Log-normal	D	Kc	7.10 × 10^9^		7.5 × 10^8^	6.6 × 10^10^
Normal	D	c	12.99	0.98	11.06	14.92
Normal	D	σ	0.65	0.04	0.58	0.72
Log-normal	I	Kc	6.42 × 10^7^		8.4 × 10^6^	4.9 × 10^8^
Normal	I	c	10.93	1.12	8.72	13.12
Normal	I	σ	0.81	0.06	0.69	0.93

*Details and explanation are in Appendix. SE, standard error; σ, population standard deviation.*

and the risk of CNS-OT is:

(5) $$Z_{I}=\left[\ln\left(K_{I}^{0.5}\right)-8.99\right)]/0.81$$

For dry exposures, the power equation and the critical *CNS-OT index* are:

(6) $$K_{D}=t^{2}\times {\mathrm{PO}}_{2}^{12.99}$$

$${\mathrm{Kc}}_{D}=7.1\times 10^{9}$$

and the risk of CNS-OT is:

(7) $$Z_{D}=\left[\ln\left(K_{D}^{0.5}\right)-11.34\right)]/0.65$$

**FIGURE 1:**
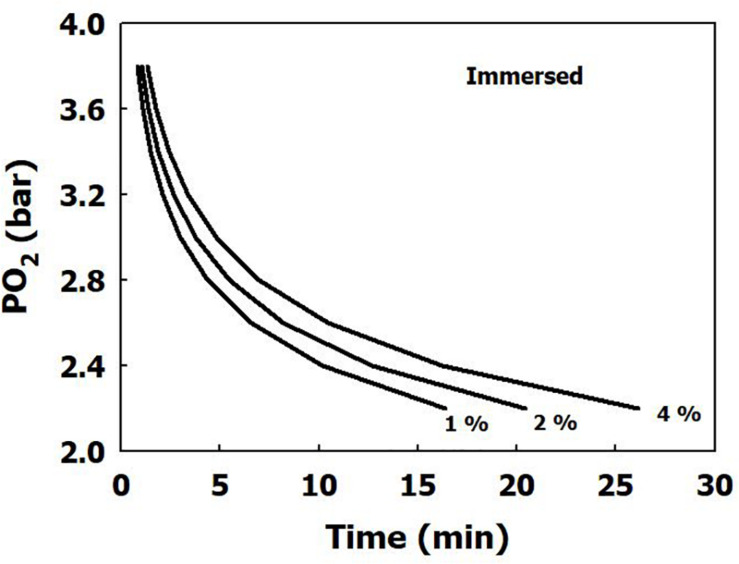
Calculated risk of CNS oxygen toxicity at 1 MET for dry exposures, as a function of time and PO_2_.

**FIGURE 2:**
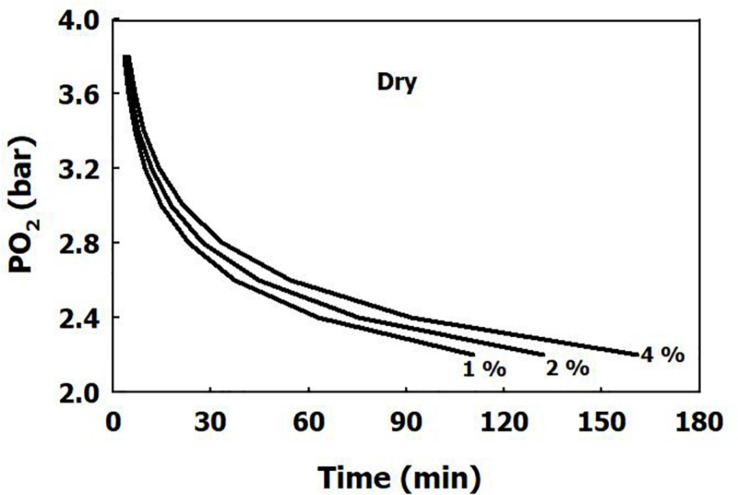
Calculated risk of CNS oxygen toxicity at 1 MET for immersed exposures, as a function of time and PO_2_.

The iso-risk lines are depicted in Figures 1, 2 for both dry and immersed exposures. It is evident that immersion carries a higher risk than dry conditions.

## Discussion

For a complex, hyperoxic exposure at rest with intervening air breaks, one may employ the previously suggested procedure (Arieli, 2019), using the same recovery function which appears as Eq. 2 in the present report.

We have previously demonstrated in the rat, that there is a linear relationship between both the power c of the PO_2_ and ln(Kc), and metabolic rate (Arieli, 2003). In the rat, the power of PO_2_ dropped from 5.90 to 2.61 as metabolic rate increased from 1 to 3 MET. In immersed humans, the power c fell from 10.93 to 6.80 with the rise in metabolic rate from 1 to 4.4 MET. The change in the power per unit metabolic rate for the rat, (2.61–5.90)/2 = −1.6, is similar to that for humans (6.80–10.93)/3.4 = −1.21. This may be indicative of a similar mechanism. We are unaware of the existence of any data for other metabolic rates in humans or other mammals. If indeed we assume the same linear relationship for humans as we noted in the rat, we may calculate the power of PO_2_ and ln(Kc) for different metabolic rates in the submerged state:

c=-1.21×MET+12.14

However, ln(Kc) was very similar at 1 and 4.4 MET, namely 17.98 and 18.02, respectively. Thus, the power equation for submerged humans as a function of time, partial pressure of oxygen, and metabolic rate would be:

$$K=t^{2}{\mathrm{PO}}_{2}^{\left(12.14-1.21\times \mathrm{MET}\right)}$$

where Kc = 6.57 × 10^7^. Nevertheless, we have no knowledge of how σ changes with metabolic rate, and therefore risk calculations are available only for 1 and 4.4 MET.

Any comparison with the effects of immersion on oxygen toxicity was usually confounded by the influence of water temperature on metabolic rate. Donald (1992) reported that at the same PO_2_ (2.5 bar), 3 of 6 divers suffered CNS-OT in dry conditions compared with 6 of 6 in the immersed state. van Ooij et al. (2011) demonstrated deterioration of lung diffusion capacity only in underwater diving (slow swimming for 3 h at 1.5 bar oxygen, in water at a temperature of 15°C and wearing a dry suit), compared with exposure in dry conditions at rest. This is the reason the limit of 2.4 bar for dry HBO exposure was reduced to only 1.3 bar in diving (Ciarlone et al., 2019).

Our present analysis has demonstrated a clear difference in sensitivity to oxygen between immersed and dry conditions (Figure 1), where thermoneutral immersion increased the risk of CNS-OT. The higher critical *CNS-OT index* (Kc) at which toxicity occurs in dry compared with immersed exposures, 7.1 × 10^9^ vs. 6.4 × 10^7^, is indeed in agreement with the increased risk of CNS-OT in submerged conditions.

A comparison with the data on CNS-OT during hyperbaric oxygen therapy is complicated by reports of convulsions, but not other symptoms related to CNS-OT, those considered in our previous study (Arieli et al., 2002) and in the present analysis. In our study of CNS-OT in closed-circuit oxygen dives (Arieli et al., 2006), the incidence of facial twitching which usually precedes convulsions was 0.2%, and loss of consciousness 0.32%, whereas the incidence of other symptoms was nausea 2.6%, dizziness 1.5%, tinnitus 0.9%, disorientation 0.6%, and tingling in the limbs 0.4%. In the present analysis of dry exposures, we found that in all of the exposures with demonstrated CNS-OT, 11.5% had either convulsions or unconsciousness and 34% had muscle twitching. Thus, convulsions are just a fraction of the symptoms related to CNS-OT.

In certain hyperbaric treatments, patients’ metabolic rate may be lower than 1 MET (for example, in carbon monoxide intoxication), which would further extend their tolerance to hyperbaric oxygen. So CNS-OT occurring during hyperbaric treatment may have an even lower incidence than that predicted by our model. For a 1 h treatment at 2.5 bar with a 10 min air break, our model predicts a 1.16% incidence of CNS-OT, whereas Welslau and Almeling (1998) reported 0.14% convulsions. For treatment on U.S. Navy Table 6, at the end of the third 20 min exposure to 2.8 bar, our model predicts a 12.7% incidence of CNS-OT. However, Smerz (2004) reported a 7.2% incidence of CNS-OT on recompression to between 2.6 and 2.9 bar. In HBO treatment of carbon monoxide poisoned patients (generally for 1 h at 3.0 bar with a 5 min air break), 5% of patients suffered seizures (Sloan et al., 1989). Although our model predicts a 9.5 or 26% risk of CNS-OT for the two protocols used to treat carbon monoxide poisoning at 2.8 bar, the incidence reported by Hampson et al. (1996) was 3%. Thus this difference will need to be taken into account in the further development of HBO therapy protocols. The considerable effect of the intervening air breaks on risk reduction may easily be calculated (Arieli, 2020), and we therefore believe that our proposed algorithm may help in the future planning of treatment tables.

## Data Availability Statement

Publicly available datasets were analyzed in this study. This data can be found here: Harabin (1993). Human central nervous system oxygen toxicity data from 1945 to 1986, NMRI Report no. 93–03. Bethesda, MD: Naval Medical Research Institute.

## Author Contributions

BA and RA contributed to the conception of the study. BA carried out data extraction. AY conducted statistical evaluation and analyzed the data. RA drafted the manuscript and prepared the figures. All authors edited and revised the manuscript, read and approved the final version.

## Conflict of Interest

AY was employed by company Indigo. The remaining authors declare that the research was conducted in the absence of any commercial or financial relationships that could be construed as a potential conflict of interest.

## Appendix

### Solution for the Parameters of the Power Equation

The power equation describes the increasing risk of CNS oxygen toxicity as *K* approaches

(8) $$K_{c}:K=t^{2}{\text{PO}}_{2}^{c}$$

From the available data, in the *i*th individual exposed to PO_2_ *i*, CNS oxygen toxicity occurs at time *t*_*i*_. There are individuals in whom toxicity does not occur, so that *t*_*i*_ may be censored. Formally, the observations are given in the following form:

(9) $$\left(y_{i},\delta_{i},{\text{PO}}_{2i}\right),i=1,\ldots ,n$$

where *y*_*i*_ = min(*t*_*i*_, *c*_*i*_) and δ_*i*_ = *I*(*t*_*i*_ ≤ *c*_*i*_), *c*_*i*_ is the censoring time, and δ_*i*_ is the indicator showing whether the observation is censored or not. The goal is to fit the model (Eq. 8) to the censored data (Eq. 9) collected from different experiments.

Considering *t* as the response variable, one can write:

$$\ln t_{i}=1/2\ln\left(K_{c}\right)-1/2c\times \ln\left({\text{PO}}_{2}\right)$$

Thus *c* and *K* can be estimated by using parametric regression techniques for the survival data. The idea is that

$$Z_{i}=\left[ln\left(t_{i}\right)+\left(c/2\right)\ln\left({\text{PO}}_{2i}\right)-\left(1/2\right)\ln\left(K_{c}\right)\right]/\sigma$$

(where σ is the population standard deviation) has some distribution *f*, where ln *t*_*i*_ can be censored. The likelihood function is written as follows:

$$\begin{array}{r} \underset{i:uncensored}{L(c,K_{c},\sigma )}=\underset{i:censored}{[\prod f\left(Zi\right)]}\;[\prod \overset{\infty }{\underset{Zi}{\int }}f(x)dx] \end{array}$$

Then −2*ln*[*L*(*c*, *K*_*c*_, σ)] is minimized over *c*, *K*_*c*_, and σ numerically. Distributions for *Z*_*i*_ can be chosen from the following list:

(1) Gaussian, *Z*_*i*_ ∼ *N*(0,1), resulting in Log-Normal distribution for T_*i*_.
(2) Smallest extreme value, resulting in Weibull distribution for T_*i*_.
(3) Logistic, resulting in Log Logistic distribution for T_*i*_, which yields a closed form expression.

In our computations, we used the Normal distribution, which demonstrated the best fit.

The risk can then be calculated from the normal distribution thus:

(10) Z=[ln⁡(t)-μ]/σ

$$\mu =E\left[\ln\left(t\right)\right]=0.5\ln\left(K_{c}\right)-\left(c/2\right)\ln\left({\text{PO}}_{2}\right)$$

where *t* is in minutes, and PO_2_ is in bar.

### Results

The equations for immersed (Eqs. 4 and 5) and dry conditions (Eqs. 6 and 7), can be mathematically transformed into the power equation.

In Immersed conditions:

$$Z_{i}=\left[\ln\left(t_{i}\right)+5.46\ln\left({\text{PO}}_{2i}\right)-8.99\right)]/0.81$$

In Dry conditions:

$$Z_{i}=[\ln(t_{i})+6.49\ln({\text{PO}}_{2i})-11.34)]/0.65$$

$$Z_{i}∼N(0,1)$$

## Abbreviations

CNS-OT: central nervous system oxygen toxicity
HBO: hyperbaric oxygen
ln: log-normal
MET: metabolic equivalent of task
PO_2_: partial pressure of oxygen.
